# Comparison of the clonality of urothelial carcinoma developing in the upper urinary tract and those developing in the bladder

**DOI:** 10.1186/2193-1801-2-412

**Published:** 2013-08-28

**Authors:** Yuding Wang, Michael R Lang, Christopher L Pin, Jonathan I Izawa

**Affiliations:** Department of Surgery, The Schulich School of Medicine and Dentistry, London Health Sciences Centre-Victoria Hospital London, The University of Western Ontario, London, ON Canada; Divisions of Pediatrics, The Schulich School of Medicine and Dentistry, London Health Sciences Centre-Victoria Hospital London, The University of Western Ontario, London, ON Canada; Divisions of Urology and Surgical Oncology, The Schulich School of Medicine and Dentistry, London Health Sciences Centre-Victoria Hospital London, The University of Western Ontario, London, ON Canada; London Health Sciences Centre-Victoria Hospital, 800 Commissioners Road East, London, ON N6A 4G5 Canada

**Keywords:** Transitional cell carcinoma, Urothelial carcinoma, Bladder cancer, Upper urinary tract cancer

## Abstract

**Purpose:**

To identify the origin of synchronous and metachronous urothelial carcinoma (UC) of the bladder and upper urinary tract to get a better understanding of the basic mechanism behind the multifocality of UC, which may provide a sound bases for the future development of new strategies for detection, prevention and therapy.

**Methods:**

Six patients with UC of the bladder and synchronous or metachronous UC of the upper urinary tract were studied. Genetic analysis involving the study of loss of heterozygosity (LOH) has been evaluated on their tumours using well characterised and new markers of UC (D9S171, D9S177, D9S303 and TP53).

**Results:**

Five of the six patients demonstrated informative results. Four of five (80%) of patients had synchronous or metacharonous UC tumour and showed patterns of LOH consistent with tumorigenesis from monoclonal tumour origin. One of five (20%) patients exhibited a LOH consistent with oligoclonal tumorigenesis.

**Conclusion:**

These findings suggest that both the monoclonal and field cancerization theory of tumorigenesis may play a role in tumors of the urothelial tract. However, more data is needed.

## Introduction

Urothelial carcinoma (UC) of the bladder is the fifth most common solid cancer in the industrialized world (Cordon-Cardo 2008). In Canada, approximately 7000 Canadians will be diagnosed with this disease (Canadian Cancer Society Cancer Statistics 2012). Approximately 30% of patients will present with de novo invasive UC and 30-40% of these patients will eventual succumb to metastatic disease (Cordon-Cardo 2008; Canadian Cancer Society Cancer Statistics 2012). UC of the upper urinary tract (UUT) is much less common, accounting for 5% of all urothelial malignancies (Kauffman & Raman 2008). Even though UC of the bladder and of the UUT originate from the same contiguous epithelial lining, primary UC of the bladder and of the UUT are biologically unique with appreciable genetic, molecular and clinical differences (Kauffman & Raman 2008). Nevertheless, UC of the bladder and of the UUT are intricately related, as these malignancies are often complicated with synchronous or metachronous malignancies of the bladder (Kang et al. 2003). Approximately 20-50% of patients with UC of the UUT will have tumour involvement in the bladder (Kauffman & Raman 2008; Kang et al. 2003). Similarly, a much smaller percentage of UC of the bladder patients go on to develop UC of the UUT (Habuchi 2005). A question of significance surrounds whether these synchronous or metachronous malignancies involving the bladder and subsequently the UUT (or vice versa) represent a clonal or oligoclonal process. The answer to this question may have significant ramifications clinically on specific cancer surveillance of the bladder and UUT, intensity of surveillance and therapeutic modalities, such as prophylactic immunotherapy or chemotherapy delivered for individual patients (Duggan et al. 2004).

There are two prospective, randomized, clinical studies that address the issue of intravesical chemotherapy to decrease the recurrence rate of UC of the bladder following a nephroureterectomy for UUT UC (Ito et al. 2013; O’Brien et al. 2011). These studies both observed that a single dose of intravesical chemotherapy following nephroureterectomy for UUT UC appeared to decease the risk of UC recurrence in the bladder. Knowing the clonality of UUT UC vs. metachronous UC of the bladder, for example, may allow for the prediction of the response to therapies, such as the intravesical chemotherapies described in these prospective trials (Ito et al. 2013; O’Brien et al. 2011). It may then be possible to more accurately determine which patients would benefit from these types of therapies vs. those in which these therapies are ineffective and can be withheld.

The clonal or oligoclonality of UC of the urothelial tract has been a hotly debated question. It is known that UC of urothelial tract commonly present as multifocal tumours (Hafner et al. 2002). The clonal explanation is known as the single transformed cell hypothesis, while the oligoclonal explanation is known as the field defect hypothesis (Hafner et al. 2002). In the single transformed cell hypothesis, it is postulated that a single cell picks up oncogenic ability and begins to proliferate and through intraluminal seeding (and implantation) or intraepithelial spread, causing multifocal tumour development (Hafner et al. 2002). Therefore, all tumours in the single transformed cell hypothesis share a common cell of origin, and thus common genetic alterations (O’Brien et al. 2011). Conversely, in the field defect hypothesis, it is postulated that a “patch” of urothelium is exposed to carcinogenic insult, and subsequently picks up oncogenic potential, with each cell within the patch acquiring individual genetic alterations (Höglund 2007). Individual cells within this patch go on to develop tumours. In this model, multifocal tumours develop from distinct cells within the urothelium (Höglund 2007).

Significant work evaluating the clonality of tumours within the urothelial tract have yielded conflicting results in favour of both the clonal and oligoclonal origin of synchronous and metachronous tumours (Hafner et al. 2002). Seminal work by Sidransky *et al.* evaluated the origin of multifocal UC of the bladder by using x-inactivation and loss of heterozygosity (LOH) as a measure of clonality (Sidransky et al. 1992). He showed that predominately multifocal tumours within the bladder show identical x-inactivation profiles (a normally random process in females) (Sidransky et al. 1992). Similarly, findings by Habuchi *et al.*, Miyo *et al.*, Xu *et al.*, and Chern *et al*. looking at p53 oncogene heterogeneity of multifocal UC of the bladder substantiated the clonal origin conclusion demonstrated by Sidransky (Habuchi et al. 1993; Miyao et al. 1993; Xu et al. 1996; Chern et al. 1996). More recent studies using an array of molecular genetic techniques as well as more specific loci for LOH studies have further added evidence to the single cell hypothesis. However, concurrent studies by Spruck *et al*., Hartmann *et al*., Takashi *et al*., and Hafner *et al*. have also shown evidence for field defect theory using similar molecular genetic techniques (LOH, and X-inactivation) (Stoehr et al. 2000; Hartmann et al. 2000; Takahashi et al. 2001; Hafner et al. 2001). Interestingly, the tumours identified showing oligoclonality were precursors or early stage tumours suggesting that UC of the bladder might be a multistep process (Takahashi et al. 2001; Hafner et al. 2001). Nevertheless, the predominate number of findings to date, suggest that most UC of the bladder to be from a single cell origin.

However, most of the clonality studies focused on synchronous or metachronous tumours involving the bladder alone, only three studies have looked at synchronous or metachronous tumours involving both the bladder and UUT (Takahashi et al. 2001; Hafner et al. 2001; Jones et al. 2005). Takahashi *et al.* analysed 34 tumours from 15 patients with UUT UC and subsequent UC of the bladder using LOH analysis on 21 microsatellite markers across eight chromosomes (Takahashi et al. 2001). It was found that 6 of 15 patients showed distinct microsatellite alteration patterns. This was in contrast to recurrent tumours within the bladder alone, evaluated in the same study, which showed only 2 of 16 patients with distinct microsatellite alteration patterns (Takahashi et al. 2001). A similar study published in the same year by Hafner *et al.* looked at 94 tumours from 19 patients with at least one tumour both within the UUT and bladder using nine markers (Hafner et al. 2001). Results showed that 5 of 19 patients showed at least two tumour clones with different genetic alterations. Interestingly, 4 of the 5 patients had UC of the bladder before UC of the ureter (Hafner et al. 2001). It is observed that UC of the UUT only occur in 0.5-2% of bladder cancer, suggesting that the tumours in these four patients were likely as arise independently (due to field defect) as opposed to intraluminal seeding (Habuchi 2005). Unfortunately, four of the five patients were male, while the last patient had non-informative loci for x-inactivation analysis. The last study by Jones *et al.* examined 58 tumours from 21 patients with 9 of the 21 patients having concurrent UUT UC and bladder UC (Jones et al. 2005). Five of nine patients showed different allelic loss patterns. Confirmation using x-inactivation analysis showed four of the five patients to exhibit random pattern of x-inactivation consistent with oligoclonality. These studies taken together suggest that oligoclonality maybe more frequent in cases with both UC of the bladder and UUT. This may be due to the fact that there are biological and clinical differences between the urothelium of the UUT and bladder (Kauffman & Raman 2008). For example, it may be possible that rapidly inactivated carcinogens may only have an effect on the UUT while exposure time to carcinogen may correlate with its carcinogenic effect on the bladder urothelium (Takahashi et al. 2001; Hafner et al. 2001). However, it is also possible that the long distance between the UUT and bladder may prevent the dominant growth of one dominant clone, which may mask oligoclonality (Hafner et al. 2002).

Animal studies using the murine (chimeric C3H/NeN-BALK/c) model exposed to known carcinogen N-butyl-N-(4-hydroxybutyl)nitrosamine has shown oligoclonal tumour development in 30% of mice, suggesting that the field defect means of tumorigenesis plays a significant role in cancer of urothelial tract (Yamamoto et al. 1998).

The three studies to date represent a small sample size. Furthermore, LOH by itself cannot confer clonality and is only as accurate as markers used in its analysis. New, more specific, loci identified to more closely match early events of tumorigenesis may offer better specificity compared to late markers of tumorigenesis, which are biased to genomic instability inherent in late cancer pathogenesis (Habuchi 2005; Hafner et al. 2002). With the use of new more specific markers coupled with previously established markers, we hope to offer new insight into nature of synchronous or metachronous UC of the bladder and UUT.

We hypothesized that synchronous and metachronous UC of the bladder and UUT will exhibit both oligoclonal and clonal characteristics pointing to a hybrid model of urothelial tumorigenesis that may be important to tumour development throughout the urothelial tract.

## Methods

### Patients

**Figure 1:**
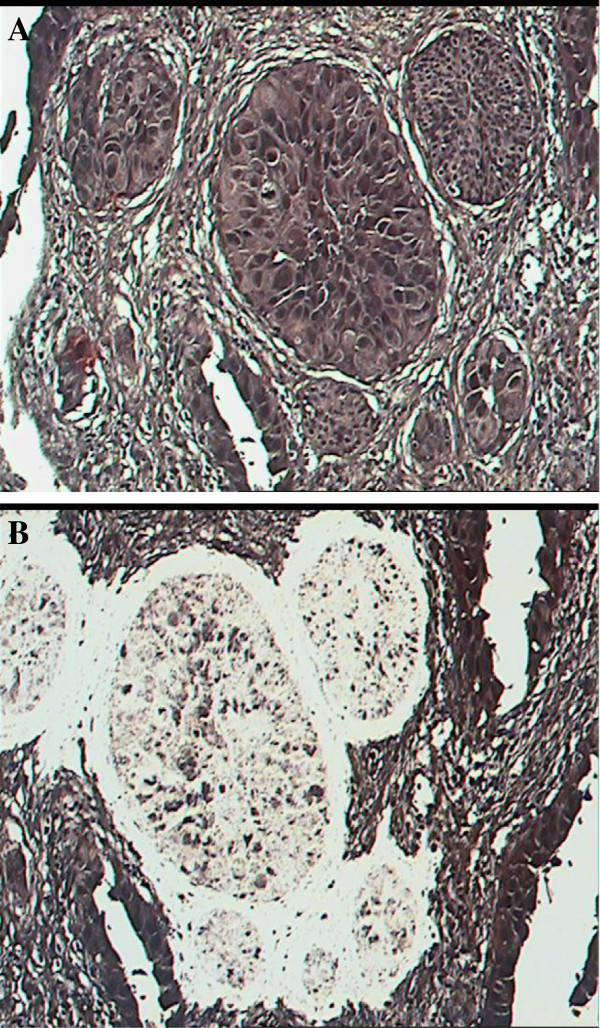
**Precise microdissection of bladder carcinoma. A)** tumour before microdissection and **B)** after microdissection.

6 patients (5 male/ 1 female) with UC of the UUT and synchronous or metachronous bladder UC that underwent surgical excision of their tumours were identified. These patients underwent nephroureterectomy for their UUT tumour(s) and transurethral resection of their bladder tumour(s). All tumours excised were high grade UC. The patients were not treated with any radiotherapy or chemotherapy prior to their surgical resection. Patients had not received any intravesical chemotherapy or immunotherapy for the bladder tumours. Tissue sample and microdissection: Formalin fixed paraffin embedded archival tissue was cut to 5um sections, and stained with haematoxylin and eosin for microscopic evaluation. Pathological identification of tumours was done with guidance of pathologist. Laser assisted microdissection of neoplastic tissue was performed using the PixCell II Laser-Capture Microdissection® apparatus (Figure 1). The exact tumour length of the entire tumour could not be determined accurately using the archival tissue. However, no tumours were so large and extensive to grow into the bladder and cause direct invasion of the bladder.

### DNA purification

DNA was isolated using a phenol/chloroform system and quantified using DNA Microdrop® quantification system. Detection of LOH: Polymerase chain reaction (PCR) was used to amplify genomic DNA at four specific loci on two different chromosomes: 9p21 (D9S171), 9q32 (D9S177), 9q22 (D9S303) and 17p13 (TP53). D9S171 is associated with tumour suppressor gene p16, a well characterized marker of early bladder tumorigenesis (Jones et al. 2005; OMIM D9S171- accessed 2010, updated updated 2010). D9S177 is associated with putative tumour suppressor gene shown in study, when altered, to be associated with one of the earliest markers of the development of UC of the bladder (OMIM D9S177- accessed 2010, updated 2009; Eguchi et al. 2010). D9S303 is associated with the PTCH proto-oncogene (Cordon-Cardo 2008; OMIM D9S303- accessed 2010, updated updated 2010). TP53 was also studied because it is a common marker of bladder tumour present in over 50% of bladder tumours (Cordon-Cardo 2008). Analysis of LOH: PCR amplification of dissected tumour and normal from the same sample was visualized using an acrylamide gel system (20%). A heterozygous normal was considered to be informative. Two independent raters evaluated the sample to assess for heterozygosity. Tumours of the UTT and bladder with the same pattern of LOH was considered from a clonal origin while those with different patterns of LOH was considered oligoclonal.

## Results

The heterozygosity of each loci (D9S171, D9S177, D9S303 and TP53) was examined in 6 patients. Of the six patients, five patients showed informative results (83%) in one of the four loci studied. However, no patient showed informative results through all four loci (Table 1).

**Table 1:** **Summery of loci evaluated of pathologically identified normal tissue of each patient case**

Patient	Microsatellite markers			
	D9S171	D9S177	D9S303	TP53
N1	-	-	-	-
N2	-	-	-	+
N3	-	-	-	+
N4	+	+	-	-
N5	-	+	-	-
N6	-	+	+	+

(+) signifies informative loci, (−) signifies non-informative loci, and (?) signifies loci that could not yet be determined.

**Figure 2:**
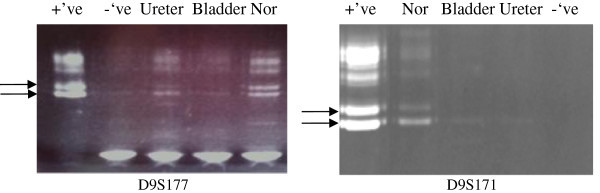
**Representative results of LOH analysis (patient 4) showing only informative loci D9S177 (left panel, and D9S171 (right panel).**

The loss of alleles as part of the tumorigenesis is a frequent event and occurred in six of seven informative loci identified in the five patients with informative loci (Table 2). Of the five patients with informative loci, only one patient (patient 4) showed a LOH pattern (loci D9S177) consistent with a divergent origin or oligoclonality (Figure 2) while the other four patients showed LOH pattern consistent with a monoclonal origin (Table 2).

**Table 2:** **Loss of heterozygosity and x-inactivation analysis of synchronous and metachronous urothelial carcinomas**

Patient	Samples	Allelic loss of heterozygosity markers			
		D9S171	D9S177	D9S303	TP53
1.	Normal	NI	NI	NI	NI
	UTT	+/−	+/+	+/+	NI
	Bladder	+/−	+/+	+/+	NI
2.	Normal	NI	NI	NI	+/+
	UTT	−/−	NI	−/−	−/−
	Bladder	+/−	NI	−/−	−/−
3.	Normal	NI	NI	NI	+/+
	UTT	NI	NI	NI	−/−
	Bladder	NI	NI	NI	−/−
4.	Normal	+/+	+/+	NI	NI
	UTT	+/−	+/+	NI	NI
	Bladder	+/−	+/−	NI	NI
5.	Normal	NI	NI	NI	NI
	UTT	NI	NI	NI	NI
	Bladder	NI	NI	NI	NI
6.	Normal	NI	+/+	+/+	+/+
	UTT	+/−	+/+	+/−	+/−
	Bladder	+/−	+/+	+/−	−/−

(+/+) heterozygosity, (+/−) LOH of the lower band, (−/+) LOH of the upper band, (−/−) complete loss of allele, (NI) non-informative results.

## Discussion

The current study provides molecular evidence for the role of both oligoclonal and monoclonal theory of tumorigenesis of synchronous and/or metachronous tumours of the urothelial tract. Four of five patients (80%) with informative loci showed a monoclonal pattern of LOH, while only one of five patients (. (20%) showed an oligoclonal pattern of LOH. Unfortunately, the patient with the oligoclonal pattern of LOH was male, and a confirmatory x-inactivation study could not be conducted. These findings are consistent with results seen by Sidranksy *et al*. (1992) Habuchi *et al*. (1993) Miyo *et al*. (1993) that show tumours of the urothelial tract occur predominately through a monoclonal process whereby a single cell is transformed causing focal tumour development and subsequently though further oncogenic insults or tumour evolution, proliferates and spreads either by intraluminal seeding (and implantation) or intraepithelial spread to cause tumours at a distant site. The observation that metastatic tumours may undergo a phase of dominancy lasting from months to years could account for latency of tumour recurrence seen within the urothelial tract (Almog 2010).

However, one patient in our study group was observed to show an LOH pattern consistent with an oligoclonal pattern of tumorigenesis explained through the field cancerization model. The two models of tumorigenesis may not be mutually exclusive. Tumour chronological experiments show that clonal evolution from a single cell may result in pseudopolyclonality in the early stages of tumorigenesis (low- grade tumours) while the overgrowth of one clone in late stages tumorigenesis (high-grade tumours) may result in pseudomonoclonality (Hafner et al. 2001). The 12 tumours studied so far from the 6 patients in this experiment have all been diagnosed as high-grade tumours. It is interesting to note that the patient with an oligoclonal pattern was from a high- grade tumour suggesting that the result seen is unlikely due to pesudopolyclonality. Our data indicates that tumour clonality of UUT UC may be a future clinical factor, among others, to determine which patients may benefit from therapies (Ito et al. 2013; O’Brien et al. 2011) to prevent UC recurrences in the bladder.

A significant number of loci identified in this study were non-informative. This was much higher than those reported in similar studies by Jones *et al.* who used similar microsatellite markers. The heterozygosity of alleles occurs in a random process and is dependent on adequate sample size to mitigate chance variation. Only four loci were evaluated in this study. Other potential loci identified in large genetic studies of urothelial cancers could be added to strengthen the power of this study. These potential loci include alleles located on 14q, 8p, 13q, and 11p showing signs of microsatellite instability present in 70%, 65%, 56%, and 54% of urothelial tumours respectively (Hartmann et al. 1999). Another potential limitation could be from the acrylamide visualization system used in this study, which may not be sensitive enough to detect minute differences in copy number variation. A potential solution may be to send results for DNA sequencing to find definitive changes between samples.

The results in this study are counter to those results seen by Takashashi *et al*. (2001) Hafner *et al*. (2001) and Jones *et al* (2005) who have also evaluated the clonality of metachronous and synchronous tumours of the UUT and bladder. Our small sample size limited loci evaluated, which may explain this difference. It is also interesting to note that the current patient population studied so far represent high-grade tumours, which are different from the tumour populations (mix of high and low grade) studied previously (Takahashi et al. 2001; Hafner et al. 2001; Jones et al. 2005).

In conclusion, the results show that most cases of UC of the UUT that occur either synchronously or metachronously to UC of the bladder seem to arise predominately from a single cell origin that spreads either through intraluminal seeding (and implantation) or intraepithelial spread. In one of case however, there was evidence for tumour that may arise independently, consistent with the field cancerization model. This suggest that although the single cell theory of tumorigenesis may be the predominate means of UC progression, the field cancerization model may also play a small albeit significant role, which must not be overlooked when considering appropriate treatments modalities, and when using molecular diagnostic techniques to diagnosis and monitor for disease recurrence.
